# SID-2 negatively regulates development likely independent of nutritional dsRNA uptake

**DOI:** 10.1080/15476286.2020.1827619

**Published:** 2020-10-12

**Authors:** Fabian Braukmann, David Jordan, Benjamin Jenkins, Albert Koulman, Eric Alexander Miska

**Affiliations:** a Wellcome Trust/Cancer Research UK Gurdon Institute, University of Cambridge, Cambridge, UK; b Department of Genetics, University of Cambridge, Cambridge, UK; c Core Metabolomics and Lipidomics Laboratory, Wellcome Trust-MRC Institute of Metabolic Science, University of Cambridge, Cambridge, UK; d Wellcome Sanger Institute, Cambridge, UK

**Keywords:** Environmental RNAi, double stranded RNA, RNA communication, nutrition, RNAi

## Abstract

RNA interference (RNAi) is a gene regulatory mechanism based on RNA-RNA interaction conserved through eukaryotes. Surprisingly, many animals can take-up human-made double stranded RNA (dsRNA) from the environment to initiate RNAi suggesting a mechanism for dsRNA-based information exchange between organisms and their environment. However, no naturally occurring example has been identified since the discovery of the phenomenon 22 years ago. Therefore it remains enigmatic why animals are able to take up dsRNA. Here, we explore other possible functions by performing phenotypic studies of dsRNA uptake deficient *sid-2* mutants in *Caenorhabditis elegans*. We find that SID-2 does not have a nutritional role in feeding experiments using genetic sensitized mutants. Furthermore, we use robot assisted imaging to show that *sid-2* mutants accelerate growth rate and, by maternal contribution, body length at hatching. Finally, we perform transcriptome and lipidome analysis showing that *sid-2* has no effect on energy storage lipids, but affects signalling lipids and the embryo transcriptome. Overall, these results suggest that *sid-2* has mild effects on development and is unlikely functioning in the nutritional uptake of dsRNA. These findings broaden our understanding of the biological role of SID-2 and motivate studies identifying the role of environmental dsRNA uptake.

## Introduction

RNA interference (RNAi) is a gene regulatory mechanism involved in many biological processes across eukaryotes [1,2]. It is mediated by the sequence specific Watson-Crick base pairing of a small regulatory RNA to a target RNA resulting in degradation, translational repression or transcriptional suppression of the target RNA [3–5]. RNAi function requires the binding of a small RNA to a protein of the Argonaute family [6]. The Argonaute guides the binding of the small RNA to the target RNA and provides the physical connection to the cellular machinery mediating gene regulation [7–9]. Overall, the interaction between a small RNA/Argonaute complex and a target RNA is the common feature of the many variations of RNAi that are found in eukaryotes.

Most commonly, RNAi is initiated and maintained by endogenously produced small RNAs such as microRNAs and piRNAs [10–12]. In addition, artificial RNA can be used to trigger RNAi. Many animals and fungi exhibit a strong RNAi response after simple addition of synthetic double stranded RNA into their environment [13–20]. The synthetic double stranded RNA is internalized by the organism and subsequently processed into small RNAs mediating gene regulation. This phenomenon is known as environmental RNAi, and is a valuable molecular biological tool used to induce sequence specific gene knock down, with important applications in medicine and agriculture [21,22].

The molecular mechanism of environmental RNAi via artificial dsRNA uptake is best understood in the model organism *Caenorhabditis elegans* [23]. A genetic screen therein identified the gene *systemic RNA interference defective-2* (*sid-2*) required specifically for environmental RNAi [24]. In this study, it was suggested that the artificial dsRNA was taken up by feeding, as the localization of SID-2 was reported to be at the apical intestinal membrane by GFP::SID-2 fusion protein experiments [24]. Later, biochemical studies showed SID-2 mediates uptake specifically of dsRNA, and single stranded RNA forming hairpins with dsRNA structure, but not the uptake of the chemically similar DNA [25]. These studies show that SID-2 is a dsRNA receptor mediating the entry of artificial dsRNA from the environment into intestinal cells.

Further studies led to mechanistic insights of dsRNA import and export downstream of *sid-2*. Biochemical and genetic studies suggest that dsRNA interaction with SID-2 triggers endocytosis at the intestinal apical membrane [24–27]. Subsequently, SID-2 is subjected to endocytic recycling involving the endosomal tyrosine kinase SID-3 and its interactor EHBP-1 [26,27]. Additional membrane trafficking is important for dsRNA transport, as highlighted by the involvement of the *trans*-Golgi network epsinR homologue RSD-3 in dsRNA import [28]. It remains unclear how dsRNA exits the endocytic pathway, however it is thought that the dsRNA channel SID-1 mediates the exit of dsRNA from an endocytic vesicle and allows the entry to the cytoplasm, where it can enter the RNAi pathway [29–32]. Furthermore, gene silencing signals can move between cells [19,33,34] and the endosome proteins SID-5 and SEC-22 are proposed to function in the export of the silencing trigger [35,36]. Overall, endomembrane transport plays an important role in dsRNA import and export, however the exact molecular mechanism remains elusive.

While much has been done on the side of the molecular mechanism, no natural occurrence of *sid-2* mediated dsRNA uptake leading to environmental RNAi has been observed. This is especially surprising because the idea that environmental dsRNA influences gene regulation was put forward more than 22 years ago [19]. Therefore, we speculated that *sid-2* might have some other function in *C. elegans* physiology.

In this work, we investigated if dsRNA uptake by *sid-2* has a dietary role by using feeding experiments in genetically sensitized mutants, and additionally asked if *sid-2* has other roles independent of dsRNA uptake by using morphological and molecular phenotyping. We do not find any evidence that *sid-2* has a dietary role, but we show that *sid-2* is a mild negative regulator of development. These results suggest *sid-2* is not a nutritional dsRNA receptor in the wild and might suggest that environmental dsRNA sampling comes with a developmental tradeoff. This work contributes to a better understanding of the biological role of *sid-2* and supports future investigations into the role of natural environmental dsRNA uptake.

## Results

### SID-2 does not enhance dsRNA uptake for nutritional reasons

In *C.elegans, sid-2* mediates intestinal uptake of artificial dsRNA that can be used to induce RNAi, however its biological function remains unknown. Interestingly, a very strong brood size reduction has been reported in a strain with mutations in *sid-2* and other genes [37]. Further, in *C. elegans*, strongly reduced brood size is also caused by the lack of nucleotides [38]. We therefore wondered whether *sid-2* takes up dsRNA for nutritional reasons.

First, we wanted to test if *sid-2* alone regulates brood size. Therefore, we analysed brood size in two independently created *sid-2* mutants. We used one backcrossed strain carrying an EMS deletion allele *sid-2(qt142)* and one carrying a novel CRISPR insertion allele *sid-2(mj465)*. Both strains showed the expected resistance to RNAi by feeding (Table 1). We measured the brood size by counting how many animals develop to adulthood for wild type and both *sid-2* mutants and represented the data using Gardner-Altman plots [39,40]. We observed a subtle reduction in brood size in only one of the two mutants. The *sid-2(qt142)* mutant showed a small but significant reduction in the number of offspring (with an estimated mean difference of 21 CI_95_ [4 to 38] fewer offspring or 90% ± 8% of wild-type levels) (Fig. S1A, Table 2). In contrast, the *sid-2(mj465)* mutant did not show a significantly reduced number of offspring (estimated mean of four fewer offspring CI_95_ [−9 to 22]) compared to wild type (Fig. S1A). This brood size analysis shows that *sid-2* has at most a small effect on brood size and suggests that *sid-2* is not essential for embryogenesis in standard laboratory conditions.

**Figure 1.**
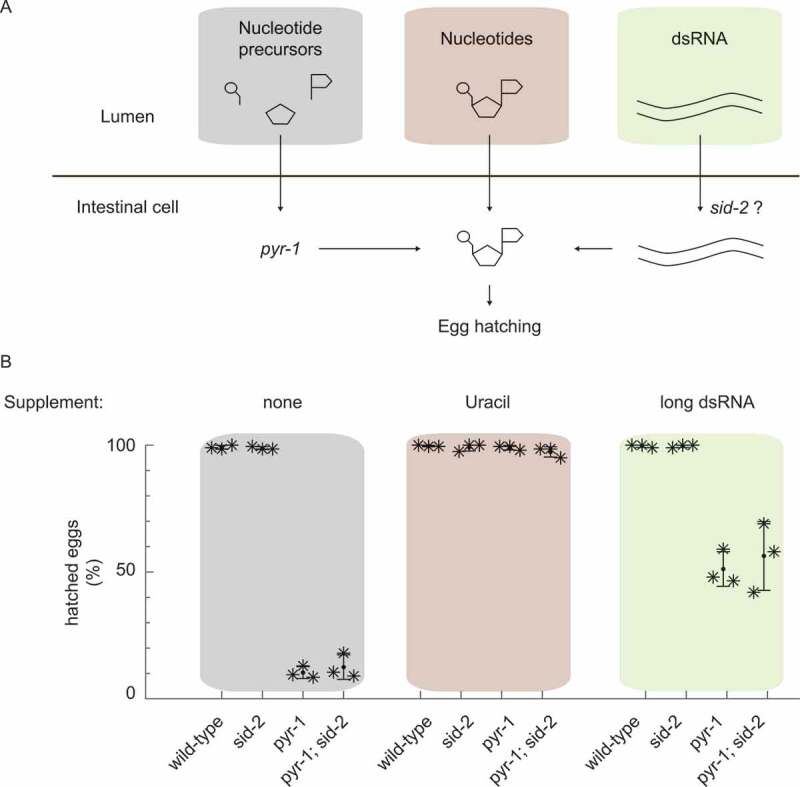
The dsRNA receptor SID-2 does not enhance dsRNA uptake for nutritional reasons

Next, we tested more directly if dsRNA uptake by *sid-2* contributes nutritionally to nucleotide levels. We designed a feeding experiment in a genetically sensitized background to uncover functions that might be masked in the nitrogen rich laboratory environment [41]. Specifically, we used a *C. elegans* strain with a compromised PYRimidine biosynthesis pathway *pyr-1* causing a strong reduction in brood size, which can be rescued with exogenous uracil [38] and we asked if exogenous double stranded RNA taken up by SID-2 can rescue this phenotype (Fig. 1A).

In this experiment, wild type, *sid-2, pyr-1* and *pyr-1;sid-2* mutants were grown on *E. coli* bacteria and one of four sources of exogenous pyrimidine (none, uracil, long dsRNA and short dsRNA). We confirmed that these strains showed the expected dsRNA uptake phenotype (Fig. S1B), and determined the hatching rate under the control condition, when no pyrimidine supplement was provided. As expected, we observed that all wild type and *sid-2* embryos hatched, while *pyr-1* mutants and *pyr-1;sid-2* double mutants showed severe embryonic development defects (Fig. 1B). Similarly, the addition of exogenous uracil rescued the embryonic development defects in *pyr-1* mutant and *pyr-1;sid-2* double mutant animals, indicating that environmental pyrimidine can contribute to nutritional value and that *sid-2* is not required for the uptake of uracil (Fig. 1B).

Finally we asked if exogenous double stranded RNA can rescue pyrimidine deficiency and if dsRNA uptake by SID-2 is required. We overexpressed long or short dsRNA in *E. coli* at levels comparable to ribosomal RNA abundance (Fig. S1C). We observed that while exogenous dsRNA was able to rescue pyrimidine deficiency, *sid-2* was not required for rescue. In *pyr-1* mutants, the expression of long or short dsRNA was able to improve the embryonic development to a hatching rate of 60% and 70%, respectively (Fig. 1B, Fig. S1D) and similar hatching rates were observed in *pyr-1;sid-2* double mutants. We propose that dsRNA uptake by SID-2 is negligible for nutritional reasons in laboratory conditions.

A) Potential sources of nucleotides contributing to egg hatching. Egg hatching depends on the availability of sufficient pyrimidines within the animal. Pyrimidines can be synthesized by *pyr-1*, taken up from the environment or potentially derived from environmental dsRNA taken up by SID-2.B) Percentage of viable eggs after pyrimidine supplementation or feeding dsRNA in wild type, *sid-2(qt142), pyr-1(cu8)* or *pyr-1(cu8); sid-2(qt142)*. Black stars represent the percentage of viable eggs of a biological replicate. Horizontal black lines indicate the mean, vertical black lines the standard deviation.

### Sid-2 *mutants display abnormal growth and morphology*

**Figure 2.**
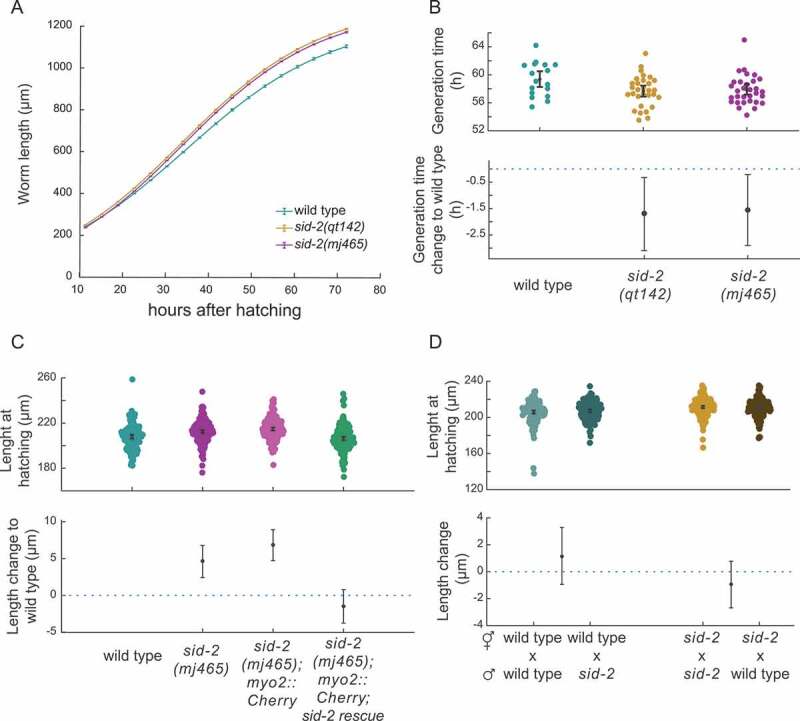
*Sid-2* mutant animals grow faster and are elongated at hatching

To discover novel functions of *sid-2*, we analysed morphological phenotypes of wild type and *sid-2* mutants. We measured single worm growth rates from late L1 to adult stage using an automated camera setup [42]. The growth rate, estimated by a logistic function, and the relative length differences indicated that the presence of SID-2 affects worm growth (Fig. 2A, Fig. S2A, Table 3). From the same worms, we calculated developmental timings. We identified a significant decrease in generation time in the *sid-2* mutants (time from egg laid until the adult animal lays its first egg) with a mean reduction of 1.7 h with a CI_95_ [−3.1 to −0.3 h] and a mean reduction of 1.6 h with a CI_95_ [2.9 to 0.2 h] (Fig. 2B). In more detail, the larvae development (from hatching to first egg) of *sid-2* mutants was significantly shorter compared to wild type, mean reduction of 1.3 h with a CI_95_ [2.4 to 0.2 h] and 1.1 h with a CI_95_ [2.2 to 0.0 h] (Fig. S2B). However, the ex utero development was not significantly different in the two mutants, mean reduction 0.2 h with a CI_95_ [0.9 to −0.4 h] and 0.3 h [0.9 to −0.3 h] (Fig. S2C). This indicated that *sid-2* affects growth rate and developmental timings.

A) Growth curve visualizing body length during development for wild type (n = 20) and *sid-2(qt142)* (n = 31), *sid-2(mj465)* (n = 33) mutants, lines represent medians and error-bars represent the 95% confidence interval of the median. B) Gardner-Altman plot for generation time of *C. elegans* wild type and indicated mutant animals. Top: Dots represent generation time for individual animals. Bottom: Dots indicate the mean difference in generation time between wild type and indicated condition. The 95% confidence interval of the mean difference is indicated by the black lines. Aggregate data from the three grow curve experiments per genotype is shown (wild type n = 17, *sid-2(qt142)* n = 30, *sid-2(mj465)* n = 31). C) Gardner-Altman plot for body length at hatching of *C. elegans* wild type and indicated mutant animals. Top: Dots represent body length at hatching of individual animals. Bottom: Dots indicate the mean difference in body length between wild type and indicated condition. The 95% confidence interval of the mean difference is indicated by the black lines. Aggregate data from at least three experiments per genotype is shown (wild type n = 150, *sid-2(mj465)* n = 196, *sid-2(mj465);myo2:Cherry* n = 184, *sid-2(mj465); myo2:Cherry;sid-2rescue* n = 182).D) Gardner-Altman plot for body length at hatching of *C. elegans* offsprings of indicated mating experiment. Top: Dots represent body length at hatching of individual animals. Bottom: Dots indicate the mean difference in body length of the test group. The 95% confidence interval of the mean difference is indicated by the black lines. Aggregate data from three experiments per cross is shown (wild type ⚥ x wild type ♂ n = 179, wild type ⚥ x *sid-2(qt142)* ♂ n = 188, *sid-2(qt142)* ⚥ x *sid-2(qt142)* ♂ n = 220, *sid-2(qt142) x* wild type ♂ n = 206)

We switched to a high magnification microscopy setup, allowing us to measure body length at birth (Fig. S2D, Table 4). We tested if *sid-2* affected the morphology of freshly hatched larvae using image analysis to measure the length at birth. The estimated mean body length at hatching for wild type was 209.5 µm, [N = 179, CI_95_ = 209.5 µm – 212.2 µm]. In contrast, the estimated mean length at hatching of two strains carrying independent *sid-2* mutant alleles is 216.9 µm, [N = 203, CI_95_ = 215.4 µm – 218.2 µm] and 216.3 µm, [N = 176, CI_95_ = 215.3 µm – 217.6 µm]. We estimated the mean increase in length for the *sid-2* mutants at 6.0 µm, [CI_95_ = 4.0 µm – 7.9 µm] and 5.4 µm, [CI_95_ = 3.4 µm – 7.2 µm], respectively (Fig. S2E). This analysis indicates that wild-type body length at hatching is shorter compared to *sid-2* mutants.

Next, we wanted to confirm that *sid-2* affects body length at hatching using a rescue experiment. Using the *sid-2(mj465)* mutant, we created two new strains, one with restored *sid-2* function via overexpression and one expressing only the selection marker as control (Fig. S2F). We found that the body length phenotype was rescued by *sid-2* overexpression. The control strain had a body length at hatching of 214.7 µm, [N = 184, CI_95_ = 213.4 µm – 216.0 µm] and the *sid-2* rescue a mean body length of 206.3 µm, [N = 182, CI_95_ = 204.9 µm – 207.8 µm]. Restoring *sid-2* function reduced mean body length by 8.3 µm [CI_95_ = 6.4 µm- 10.4 µm] compared to control indicating that *sid-2* functions in regulation of body length. The body length in *sid-2* rescue was similar to wild type by −1.5 µm [CI_95_ = −3.8 µm – 0.8 µm] (Fig. 2C). These experiments confirm that *sid-2* affects body length at birth.

Because *sid-2* can take up dsRNA from the environment, we wanted to know if additional environmental dsRNA affects body length. We measured the length at hatching of wild type and *sid-2* mutants fed on dsRNA-overexpressing or control bacteria. We observed that *sid-2* mutants were longer than wild type whether dsRNA was overexpressed or not. In control conditions, *sid-2* mutant body length was 7.5 µm, CI_95_ [5.7 µm – 9.3 µm] longer compared to wild type. A similar increase was observed in the presence of dsRNA, 6.5 µm, CI_95_ [5.0 µm – 8.1 µm] (Fig. S2G) suggesting that the body length change in *sid-2* mutants was robust to environmental dsRNA.

Furthermore, we tested if genes that are involved in RNAi and function downstream of *sid-2* share a similar phenotype. We measured the length at hatching of the dsRNA binding protein mutant *rde-4* and two independent mutants of the dsRNA transported *sid-1*. We observed that *rde-4* mutants showed in contrast to *sid-2* mutants a shorter length at hatching CI_95_ [−4.9 µm – 0.5 µm] than wild type and that *sid-1* did not affect worm length significantly (*sid-1(qt129)* CI_95_ [−0.1–4.1 µm] and *sid-1(mj444)* CI_95_ [−2.5 µm – 1.5 µm]) (Fig. S2H, Table 1) suggesting that the downstream processes of RNAi are not involved in *sid-2* body length change.

Next, we asked if the increase in body length due to *sid-2*’s function is embryonic or maternally inherited. Therefore, we performed mating experiments restoring *sid-2* function within an embryo. We observed that larvae from *sid-2* mutant mothers mated with restored *sid-2* function were as long as larvae without restored *sid-2* function indicating that body length regulation by *sid-2* is maternally inherited (Fig. 2D). Overall these experiments indicate that *sid-2* affects worm morphology.

### Sid-2 *is required for the normal molecular composition of embryos and adults*

**Figure 3.**
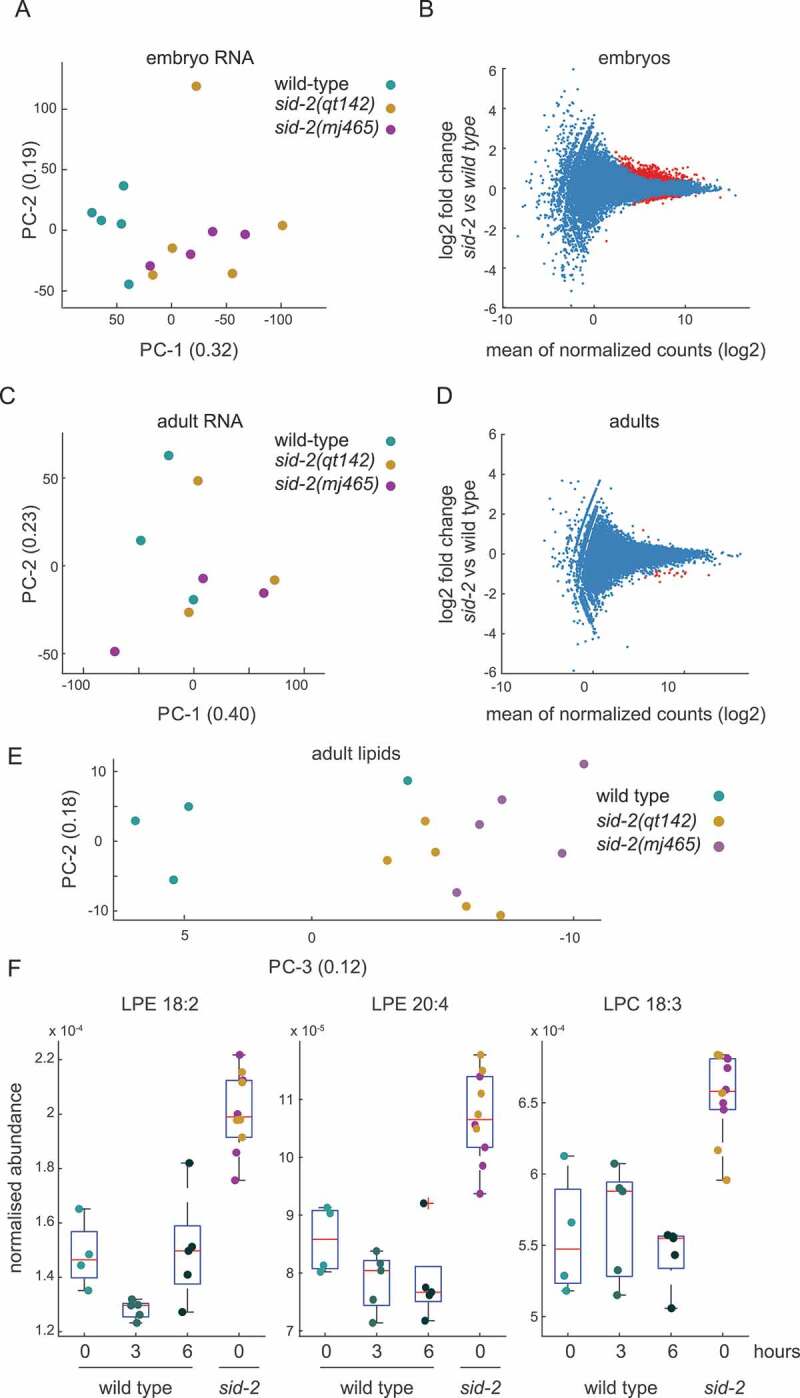
*Sid-2* mutants show molecular phenotypes at embryonic and adult stage

Having observed developmental effects of *sid-2*, we wanted to identify molecular phenotypes in *sid-2* mutants. First, we profiled the transcriptome of embryos collected five days after L4 were transferred, from *sid-2* and wild-type mixed populations. Using principal component analysis allowed us to seperate *sid-2* and wild type in the first (0.32) principal component (Fig. 3A). To describe the difference in the transcriptomes, we performed differential expression analysis and identified that 73 genes were significantly differentially expressed (FDR < 0.01 and > two-fold difference) (Fig. 3B, Table 5). A gene enrichment analysis for significantly differentially expressed genes identified a significant enrichment for phenotypic processes including larva, dauer and fat physiology (FDR < 0.05 and ≧ two observations) supporting the idea that *sid-2* embryos are significantly different (Fig. S3A). Furthermore, we embedded our transcriptome data into a pseudotime line of transcriptome data [43]. This analysis placed the *sid-2* mutant transcriptome with more mature embryos compared to the wild type transcriptome (Fig. S3B). Together, this analysis indicates that *sid-2* embryos were slightly ahead in development.

A) Principal component analysis representing the embryo transcriptome along the first and second principal component of wild type (n = 5) and *sid-2* mutants (total n = 9), *sid-2(qt142)* n = 4, *sid-2(mj465)* n = 5). Individual circles represent aggregated transcriptome data of individual biological replicates. B) MA plot visualizing embryo transcriptome comparison of wild type (n = 5) and *sid-2* mutants (total n = 9, *sid-2(qt142)* n = 4, *sid-2(mj465)* n = 5). Each red circle represents a statistically significant (DE) transcript (FDR < 0.01).C) Principal component analysis representing the adult transcriptome along the first and second principal component of wild type (n = 3) and *sid-2* mutants (total n = 3), *sid-2(qt142)* n = 3, *sid-2(mj465)* n = 5). Individual circles represent aggregated transcriptome data of individual biological replicates. D) MA plot visualizing adult transcriptome comparison of wild type (n = 3) and *sid-2* mutants (total n = 6, *sid-2(qt142)* n = 3, *sid-2(mj465)* n = 3). Each red circle represents a statistically significant (DE) transcript (FDR < 0.01). E) Principal component analysis representing the adult lipid metabolome along the second and third principal component of wild type (n = 4) and *sid-2* mutants (total n = 10), *sid-2(qt142)* n = 5, *sid-2(mj465)* n = 5). Individual circles represent aggregated lipid metabolome data of individual biological replicates. F) Boxplot of lipid metabolites that are significantly different between *sid-2* mutant (total n = 10, *sid-2(qt142)* n = 5, *sid-2(mj465)* n = 5) and wild type (n = 4–5) samples at all three timepoints (50, 53 and 56 hours after L1 starvation) (T-test, FDR < 0.05). Individual circles represent lipid levels of individual biological replicates. LPE: lysophosphatidylethanolamine, LPC: lysophosphatidylcholine.

Next, we aimed to identify molecular phenotypes in adult animals, reasoning that *sid-2* function in adult animals translates to the maternally inherited effect on embryos. First, we analysed the transcriptome of young adult animals. *Sid-2* mutants and wild type were not separated into distinguished groups using any combinatorial pair of the first four principal components indicating that adult transcriptomes are more similar than embryo transcriptomes (Fig. 3C). Next, we performed differential expression analysis and identified nine genes significantly differentially expressed genes in *sid-2* mutant (FDR < 0.01 and > two-fold difference) compared to wild type (Fig. 3D). Finally, we performed a gene enrichment analysis, however no significant enrichment for phenotypic processes was identified (FDR < 0.05 and ≧ two observations). Overall, this transcriptome analysis indicates that *sid-2* and wild type transcriptomes are similar in adults.

Since, we detected transcriptome changes for genes involved in fat physiology, we performed a time course lipid metabolomic analysis. We sampled *sid-2* mutant and wild type 50 hours after synchronized L1 were plated. At this developmental time point, *sid-2* mutant and wild type animals are at the young adult stage. In addition, we collected wild type samples three and six hours later. In the lipid metabolomic analysis, 257 out of 397 lipids were detected in all samples and analysed further (Table 6). First, we performed principal component analysis and were able to distinguish *sid-2* mutants and wild type collected at 50 hours with a combination of the second (0.18) and third (0.12) principal component (Fig. 3E), but not on the first (0.33) and second (0.18) principal component (Fig. S3C) indicating that lipids differ between wild type and *sid-2* mutants. To identify effects on lipid composition of *sid-2*, we applied t-test analysis on normalized lipid metabolome data with multiple testing correction (FDR < 0.05). We identified a total of nine *sid-2* dependent lipids with significantly different abundance. All *sid-2* dependent lipids are hydrolyzation products of phospholipids of the enzymes phospholipase A2 class and are either part of the lysophosphatidylethanolamine (four) or lysophosphatidylcholine (five) class [44–47]. These lysophospholipids have diverse functions including cell signalling and growth regulation in the mammalian system [48,49] and show dynamic abundance during worm development [50]. Interestingly, two lysophosphatidylethanolamine (LPE 18:2 and LPE 20:4) and one lysophosphatidylcholine (LPC 18:3) were significantly more abundant in *sid-2* mutants compared to wild type at all time points (Fig. 3F). The other two lysophosphatidylethanolamine and four lysophosphatidylcholine were significantly higher in *sid-2* mutants if compared to wild type at 50 hours, but were indifferent at wild type 53 hours and/or wild type 56 hours (Fig. S3D). We did not observe any significant difference in energy storage lipids like triglycerides (Table 6) suggesting that *sid-2* does not affect energy storage but affects some deacetylated phospholipids. Overall, the feeding experiments together with the morphological and molecular profiling provide the first experimental data indicating that *sid-2* mediated dsRNA uptake is not nutritional and uncover an unexpected role of *sid-2* in slowing down development.

## Discussion

In *C. elegans*, SID-2 can take up artificial dsRNA from the environment which induces extremely potent gene regulation and results in a variety of phenotypic changes. Here we explore alternative functions of *sid-2*, inspired by the lack of natural examples of direct gene regulation by environmental RNAi. We first experimentally address the possibility of a dietary role of dsRNA and do not find evidence for SID-2 dependent dsRNA uptake providing a source of nutrition. In addition, using quantitative phenotyping and RNA sequencing, we discovered a novel role of *sid-2* in slowing development. Here we argue against a dietary role of *sid-2*, and discuss the novel effect of *sid-2* in development. These findings provide guidance for future studies investigating *sid-2* function and the role of environmental dsRNA uptake in a more natural context.

### *No indication for nutritional dsRNA uptake by* sid-2

Several of our experiments help to understand if *sid-2* functions in nutrition. We looked at a contribution to nucleotide supply in the worm and we examine developmental rate and lipid metabolism for signs of malnutrition in *sid-2* deficient worms. We found in our feeding assay that *sid-2* was not required for rescue, however environmental dsRNA rescues nucleotide deficiency. How does the environmental dsRNA contribute to nutrition independent of *sid-2*? Environmental dsRNA might be degraded in the intestine by digestive enzymes and allowing the uptake of these products through intestinal nucleoside transporters [51,52]. Additional research is required to determine the nature of alternate mechanisms.

Further, our development rate analysis supports that *sid-2* deficient worms are not malnourished. While impaired feeding behaviour or limited nutrients usually slow development [53–55], we observed growth rate and generation times consistent with accelerated development in *sid-2* mutants. This did not match a signature of dietary restriction. Similarly, in our lipid metabolome analysis *sid-2* mutants showed unaffected levels of energy storage lipids and increased levels of lysophosphatidylcholines and lysophosphatidylethanolamines. These three lipid classes show decreased levels in nutrient deprived *C. elegans* [56]. Thus, the lipid profiling, the feeding experiments and the developmental analysis suggest that dsRNA uptake by *sid-2* has no nutritional role.

### Development rate a trade off for primary function?

Animal body length is affected by genes, environment and development, and probably under strong evolutionary selection [57–60]. Traditional genetic screens have identified genes that dramatically increase adult body length (double). Evolutionary analyses suggest that such changes in body length in nematodes is usually mediated by changes in cell size rather than cell number [61,62]. Molecularly, these genes alter body length through modulation of endoreplication in hypodermal cells and the molecular composition of the extracellular cuticle produced by the hypodermis [62,63]. Similarly, larval body length is also plastic, but more subtly affected by genes and the environment. Larva body length is altered by up to ±10% by strong dietary restriction, maternal age, and by Insulin receptor *daf-2* (abnormal *da*uer *f*ormation) [64–66]. The decrease in larvae body length by *sid-2* is comparable to decreases in such conditions. How *sid-2* affects body length and if dsRNA is involved remains to be investigated. It is possible that the body length reduction by *sid-2* is a trade off for another function such as environmental RNAi.

### *Towards an understanding of a conserved function of* sid-2

We believe additional phenotypic studies in combination with evolutionary analysis could complete our understanding of *sid-2’s* effect on development. In many *Caenorhabditis* species a *sid-2* homologue is present, but *sid-2* has lost the ability to take up dsRNA for environmental RNAi in many closely related species [24,25,67]. For example, the sister species *C. briggsae* and *C. remanei* are resistant to environmental RNAi even though a homologue of *sid-2* is present. In both species, the transgenic expression of *C. elegans sid-2* renders them susceptible to environmental RNAi [24,67] indicating either that their endogenous *sid-2* doesn’t have the ability to take up dsRNA or that the dsRNA taken up by their endogenous *sid-2* is not made accessible to the downstream machinery required for environmental RNAi [24,25,67]. Whether *sid-2* has a conserved effect on development can be understood by generating *sid-2* mutants in other *Caenorhabditis* species and assessing their development. Further, whether dsRNA is involved in such an effect could be addressed by leveraging the existing knowledge of dsRNA uptake deficiency and competence in *Caenorhabditis* species [24,67]. This analysis could be extended to animals with the ability to take up dsRNA from the environment and the western corn rootworm presents an ideal starting point, since dsRNA uptake mutants already exist and it is of agricultural importance [68].

We envision that environmental dsRNA uptake does exist as a biological mechanism and as a role of *C. elegans sid-2*. Experimental evidence suggests a RNAi based mechanism targeting a single gene, however past research failed to identify such a mechanism [69,70]. We believe alternative hypotheses are worth exploring. For example, we could imagine that *sid-2* is an environment reader. Instead of leading to sequence specific gene expression changes of a specific gene, SID-2 could sample environmental dsRNA to store information, possibly creating an ‘RNA fingerprint’ of *C. elegans’* bacterial environment in the wild [71–73]. Potentially, the resulting smallRNA fingerprint could serve as an RNA-based memory and by an unknown mechanism be associated with an environmental condition to inform future decision making. Such a mechanism could be involved in the ability of *C. elegans* to develop aversion to natural and artificial bacterial stressors [74,75]. This hypothesis could be tested with an associated learning assay using dsRNA and *sid-2* mutants.

Overall, we discover *sid-2* affects development independently of artificial dsRNA uptake, and find that it does not enhance nutritional uptake of dsRNA. These findings will help to understand the biological role of SID-2 and motivate studies identifying the role of environmental dsRNA in RNA communication.

## Methods

### Nematode culture and strains

*C. elegans* was grown under standard conditions at 20 °C. Bristol N2 was used as wild-type strain [61]. The *E. coli* strain HB101 *[supE44 hsdS20(rB-mB-) recA13 ara-14 proA2 lacY1 galK2 rpsL20 xyl-5 mtl-1]* was used as food source, except HT115 [F-, mcrA, mcrB, IN(rrnD-rrnE)1, rnc14::Tn10(DE3) lysogen: lavUV5 promoter -T7 polymerase] was used in the pyrimidine supplement experiment and RNAi experiments. Both bacteria strains were obtained from the Caenorhabditis Genetics Centre, University of Minnesota, Twin Cities, MN, USA.

### RNAi experiments

Empty vector, *pos-1* and *rpb-2* bacterial feeding clones were a kind gift from J. Ahringer’s laboratory. Bacteria were grown in LB Ampicillin for 6 hours, then seeded onto 50 mm NGM agar plates containing 1 mM IPTG and 25 μg/ml Carbenicillin. A volume of 200 μl bacterial culture per plate was used and left to dry for 48 hours. L1 larvae were synchronized by bleaching and transferred onto RNAi plates. Their body length compared to wild type was assayed after 72 hours. For RNAi of *pyr-1* mutant strains, 0.5% uracil was added to the NGM IPTG-Carb plates.

### Pyrimidine supplementation assay

IPTG-CARB NGM plates were seeded with HT115 bacteria carrying either a plasmid (pR70 (none), L4440 (short) or GPF (long)). For the uracil condition, IPTG-CARB NGM plates were supplemented with 0.5% uracil according to [38] and were seeded with bacteria carrying the plasmid without T7 sides. Animals were grown to adulthood and transferred for egg laying to a new plate. On the next day, 100 hundred eggs were transferred to a new plate and 24 hours later the number of hatching eggs were counted.

### Molecular cloning

Plasmid pR70 for bacteria expression ‘none’ was cloned from L4440 using Gibson cloning resulting in a plasmid in which both T7 promoters and the Multiple cloning site was removed. Cloning was performed using a home made Gibson mix using the NEB Gibson Assembly protocol (E5510) using the primer GCCAGCGATAAGCCAGGTTGCTTCC and TATCGCTGGCGTTACCCAACTTAATC. A gene product from IDT corresponding to the SID-2 genomic sequence starting from 980 bp upstream of SID-2 start codon to 119 bp after the stop codon was cloned into a linearized pUC19 with the primers caagcaattttttgatatatGGTCGACTCTAGAGGATC and tcaataaagatcttgtgagtTGCAGGCATGCAAGCTTGG. Plasmids were transformed into DH5ɑ (Bioline, BIO-85,026) and purified using PureLink HQ Mini Plasmid DNA (Life Technologies Ltd, K210001).

### RNA quantification

RNA was isolated from bacteria using TRIZOL and quantified using Qubit RNA BR Assay Kit (Life technology, Q10210). To quantify over-expressed RNA, 50 ng of total RNA was size separated using RNA ScreenTape Analysis (Agilent, 5067–5576) and quantified using TapeStation software.

### Mutagenesis

CRISPRCas9 genome editing was performed with homemade Cas9 and gRNA, crRNA from Dharmacon using the concentrations indicated in (Table 7) according to [76]. *C. elegans* SX3237*sid-2(mj465)* were injected to generate the *sid-2* rescue strain SX3432 *sid-2(mj465) III; mjEx597[myo2::mCherry;sid-2genomic]* and the control strain SX3432 *sid-2(mj465) III;mjEx596[myo2::mCherry].*

### Brood size measurement

Brood size measurements were completed over three 24-h intervals. Individual L4 animals were transferred to a new plate (day 0). For 3 days, each day (days 1–3), each animal was transferred to a new plate. The eggs were allowed to hatch and grow for 3 days and the number of animals was counted.

### Development and phenotype analysis

Growth curves were estimated from long-term video imaging. To obtain synchronized embryos, 20 L4 animals were transferred to a new HB101 NGM plate. After 24 hours, the now adult animals were moved to a new HB101 NGM plate and allowed to lay eggs for 1 h. Next, individual eggs were transferred to imaging plates. A custom camera system was used to record back-lit images through the development from the ex utero egg stage to the egg-laying adult stage (≈ 65 h). To accomplish this, an imaging system was built with a robot arm mounted camera (Flea3 3.2MP monochrome, Point Grey) moving between wells to record images sequentially. Each well contained at the start a single *C. elegans* embryo nematode and every ≈ 3 min a picture of the animals were recorded for ≈ 3 days. The resulting movies were analysed off-line with a custom written MATLAB script (Mathworks) to calculate a growth curve estimated by a logistic function (logistic max, logistic rate, logistic shift). For the relative length analysis, bootstrap samples of the length ratios of the mutant and the wild type were collected for 15 windows with a length of 4 h and 16 min starting at 0 h. If the real-time difference between the two samples in a given ratio was greater than 1.5 h this ratio was rejected. One thousand bootstrap samples were collected in this way for each mutant at each time point, and the mean and 95% CI of the ratio was computed. Ex-utero development time was calculated using the time adults were moved to HB101 plate and the time of hatching extracted from recorded images. Similarly, time from hatching until the first egg was calculated using the time of hatching and the time of the appearance of the first laid egg extracted from recorded images. Likewise, generation time was calculated using the time adults were moved to HB101 plate and the time of the appearance of the first laid egg extracted from recorded images.

### Imaging plates

Imaging plates for developmental analysis were made using the standard NGM recipe, but without peptone and cholesterol. Furthermore, agarose was substituted with 0.8% Gelzan (Sigma G1910-250 G) for a more transparent gel. Imaging plates were seeded with one µL of HB101 concentrate at optical density 20.

### L1 length analysis

To obtain synchronized embryos for L1 length analysis, 40 L4 animals were transferred to a new HB101 NGM plate. After 24 h, the now adult animals were moved to a new HB101 NGM plate and allowed to lay eggs for 1 h. ~100 eggs were transferred on NGM plate without food and imaged with a leica DM6B microscope. The resulting movies were analysed off-line with a custom written MATLAB script (Mathworks). In short, animals were segmented from the background using an intensity threshold and a skeleton was extracted using the *bwmoprh* function. The length of the skeleton was computed using the *bwdistgeodesic* function.

### RNA library preparation

Embryo RNA seq: Ten L4 animals were grown for 5 days at 20 °C. After washing thoroughly with M9 to remove bacteria, eggs were isolated using bleach, washed in M9 and resuspended in TRIsure (Bioline, BIO-38,033). Young adult seq: Synchronized L1 were grown for 50 hours. After washing thoroughly with M9, the samples were resuspended in TRIsure. Eggs and young adult samples in TRIsure were lysed with five freeze-thaw cycles in liquid nitrogen. Total RNA was isolated by chloroform extraction. Ribosomal RNA was depleted from total RNA using NEBNext rRNA Depletion (NEB, #E6350) and libraries prepared using NEBNext Ultra™ II Directional RNA Library Prep Kit for Illumina (NEB, #E7760). Libraries were sequenced on Illumina HiSeq 1500.

### Bioinformatic analysis

RNA reads were aligned using STAR against the *C. elegans* genome WS235 [77]. Read counts per genetic element of the Wormbase genome annotation WS235 were calculated using feature counts [78]. Reads were normalized using pseudoreference with geometric mean row by row [79] and statistical analysis was performed using Benjamini-Hochberg (BH) adjustment [80] using the MATLAB (Mathworks) function ‘nbintest’ with the ‘VarianceLink’ setting ‘LocalRegression’. Embryonic time course RNA sequencing data was obtained from NCBI Sequence Read Archive [43].

### Gene ontology analysis

Significantly differentially expressed genes (FDR < 0.01 and >2 fold change) between wild type and *sid-2* were used as input for the enrichment analysis of wormbase.org [81].

### Principal components analysis of RNA sequencing data

Normalized read counts from embryonic *sid-2* mutant and wild type RNA sequencing data were log transformed and centred. Next, the covariance matrix and the first 10 eigenvectors were calculated using the function eigs of MATLAB (Mathworks) for the Boeck et al. embryonic RNA sequencing data alone. The projection onto the first 10 eigenvectors was calculated for the centred data of all samples.

### LC/MS lipid profiling

*Sid-2* mutant and wild type young adults were sampled 50 h after synchronized L1 were plated. Additional wild type samples were collected three and 6 h later.

*C. elegans* were prepared for LC-MS lipidomics as previously described with minor modifications [82]. Briefly, ~1000 *C. elegans* worms were re-suspended in 100 µL of water, then 0.4 mL of chloroform was added to each sample followed by 0.2 mL of methanol. The samples were then homogenized by vortexing then transferred into a 2 mL Eppendorf screw-cap tube. The original container was washed out with 0.4 mL of chloroform: methanol (2:1, respectively) and added to the appropriate 2 mL Eppendorf screw-cap tube. This was followed by the addition of 150 µL of the following stable isotope labelled internal standards (approximately 10 to 50 µM in methanol): Ceramide(C16d31), LPC(C14:0d42), PC(C16:0d31/C18:1), PE(C16:0d31/C18:1), PG(C16:0d31/C18:1), PI(C16:0d31/C18:1), PS(C16:0d62), SM(C16:0d31) and TG(45:0d29). Then, 400 µL of sterile water was added. The samples were vortexed for 1 min and sonicated for 30 min, and then centrifuged at ~20,000 rpm for 5 min. The organic layer (the lower chloroform layer) was collected into a 2 mL amber glass vial (Agilent Technologies, Santa Clara California, USA) and dried down to dryness in an Eppendorf Concentrator Plus system (Eppendorf, Stevenage, UK) run for 60 min at 60°C. The dried lipid samples were then reconstituted with 100 µL of 2:1:1 solution of propan-2-ol, acetonitrile and water, respectively, and then vortexed thoroughly. The lipid samples were then transferred into a 300 μL low-volume vial insert inside a 2 mL amber glass auto-sample vial ready for LC-MS analysis of intact lipid species.

Full chromatographic separation of intact lipids was achieved using Shimadzu HPLC System (Shimadzu UK Limited, Milton Keynes, United Kingdom) with the injection of 10 µL onto a Waters Acquity UPLC® CSH C18 column; 1.7 µm, I.D. 2.1 mm X 50 mm, maintained at 55°C. Mobile phase A was 6:4, acetonitrile and water with 10 mM ammonium formate. Mobile phase B was 9:1, propan-2-ol and acetonitrile with 10 mM ammonium formate. The flow was maintained at 500 µL per minute through the following gradient: 0.00 minutes_40% mobile phase B; 0.40 minutes_43% mobile phase B; 0.45 minutes_50% mobile phase B; 2.40 minutes_54% mobile phase B; 2.45 minutes_70% mobile phase B; 7.00 minutes_99% mobile phase B; 8.00 minutes_99% mobile phase B; 8.3 minutes_40% mobile phase B; 10 minutes_40% mobile phase B. The sample injection needle was washed using 9:1, 2-propan-2-ol and acetonitrile. The mass spectrometer used was the Thermo Scientific Exactive Orbitrap with a heated electrospray ionization source (Thermo Fisher Scientific, Hemel Hempstead, UK). The mass spectrometer was calibrated immediately before sample analysis using positive and negative ionization calibration solution (recommended by Thermo Scientific). Additionally, the heated electrospray ionization source was optimized at 50:50 mobile phase A to mobile phase B for spray stability (capillary temperature; 380°C, source heater temperature; 420°C, sheath gas flow; 60 (arbitrary), auxiliary gas flow; 20 (arbitrary), sweep gas; 5 (arbitrary), source voltage; 3.5 kV. The mass spectrometer resolution was set to 25,000 with a full-scan range of m/z 100 to 1,800 Da, with continuous switching between positive and negative mode. Lipid quantification was achieved using the area under the curve (AUC) of the corresponding high resolution extracted ion chromatogram (with a window of ± 8 ppm) at the indicative retention time. The lipid analyte AUC relative to the associated internal standard AUC for that lipid class was used to semi-quantify and correct for any extraction/instrument variation.

PCA of Lipid metabolites were performed as follows. First, undetected lipid metabolites were removed (lipid metabolite detected in less than half +1 of the samples). Next, a small number (+0.000001) was added to the lipid metabolite data. Then, normalized lipid metabolite data were log10 transformed and converted to z-scores. Next, the covariance matrix of the data and the first 10 eigenvectors were calculated using the function eigs of MATLAB (Mathworks). The projection onto the first 10 eigenvectors was calculated for the centred data of all samples.

Significant differences in lipid metabolite composition between wild type and *sid-2* mutants were detected as followers. First, undetected lipid metabolites were removed (lipid metabolite detected in less than half +1 of the samples) and normalized by the total amount of detected lipids. Then, two-sample t-tests were performed testing the H0: The data of wild type and *sid-2* mutant comes from independent random samples with equal means and the H1: That the population means are not equal using the MATLAB function ttest2 with the Tail:both setting. Subsequently, the positive false discovery rate was estimated for multiple hypothesis testing using the testing correction based on Benjamini and Hochberg [80] using the MATLAB function mafdr.

## Supplementary Material

Supplemental Material

## Data Availability

Sequencing data is available in the European Nucleotide Archive under the study accession number PRJEB32813.

## References

[1] Cerutti H, Casas-Mollano JA. On the origin and functions of RNA-mediated silencing: from protists to man. Curr Genet. 2006;50(2):81–99. 16691418 10.1007/s00294-006-0078-x PMC2583075

[2] Shabalina SA, Koonin EV. Origins and evolution of eukaryotic RNA interference. Trends Ecol Evol. 2008;23(10):578–587. 18715673 10.1016/j.tree.2008.06.005 PMC2695246

[3] Ghildiyal M, Zamore PD. Small silencing RNAs: an expanding universe. Nat Rev Genet. 2009;10:94–108. 19148191 10.1038/nrg2504 PMC2724769

[4] Siomi H, Siomi MC. On the road to reading the RNA-interference code. Nature. 2009;457(7228):396–404. 19158785 10.1038/nature07754

[5] Wilson RC, Doudna JA. Molecular mechanisms of RNA interference. Annu Rev Biophys. 2013;42(1):217–239. 23654304 10.1146/annurev-biophys-083012-130404 PMC5895182

[6] Meister G. Argonaute proteins: functional insights and emerging roles. Nat Rev Genet. 2013;14:447–459. 23732335 10.1038/nrg3462

[7] Sheu-Gruttadauria J, MacRae IJ. Structural foundations of RNA silencing by argonaute. J Mol Biol. 2017;429(17):2619–2639. 28757069 10.1016/j.jmb.2017.07.018 PMC5576611

[8] Peters L, Meister G. Argonaute proteins: mediators of RNA silencing. Mol Cell. 2007;26(5):611–623. 17560368 10.1016/j.molcel.2007.05.001

[9] Hutvagner G, Simard MJ. Argonaute proteins: key players in RNA silencing. Nat Rev Mol Cell Biol. 2008;9(1):22–32. 18073770 10.1038/nrm2321

[10] Bartel DP. Metazoan MicroRNAs. Cell. 2018;173(1):20–51. 29570994 10.1016/j.cell.2018.03.006 PMC6091663

[11] Weick E-M, Miska EA. piRNAs: from biogenesis to function. Development. 2014;141(18):3458–3471. 25183868 10.1242/dev.094037

[12] Chapman EJ, Carrington JC. Specialization and evolution of endogenous small RNA pathways. Nat Rev Genet. 2007;8(11):884–896. 17943195 10.1038/nrg2179

[13] Nowara D, Gay A, Lacomme C, et al. HIGS: host-induced gene silencing in the obligate biotrophic fungal pathogen blumeria graminis. Plant Cell. 2010;22(9):3130–3141. 20884801 10.1105/tpc.110.077040 PMC2965548

[14] Koch A, Kumar N, Weber L, et al. Host-induced gene silencing of cytochrome P450 lanosterol C14α-demethylase–encoding genes confers strong resistance to Fusarium species. Proc Nat Acad Sci. 2013;110(48):19324–19329. 24218613 10.1073/pnas.1306373110 PMC3845197

[15] Baum JA, Bogaert T, Clinton W, et al. Control of coleopteran insect pests through RNA interference. Nat Biotechnol. 2007;25:1322. 17982443 10.1038/nbt1359

[16] Mulot M, Boissinot S, Monsion B, et al. Comparative analysis of RNAi-based methods to down-regulate expression of two genes expressed at different levels in Myzus persicae. Viruses. 2016;8(11):316. 10.3390/v8110316 PMC5127030 27869783

[17] Wang M, Weiberg A, Lin F-M, et al. Bidirectional cross-kingdom RNAi and fungal uptake of external RNAs confer plant protection. Nat Plants. 2016;2(10):16151. 27643635 10.1038/nplants.2016.151 PMC5040644

[18] Koch A, Biedenkopf D, Furch A, et al. An RNAi-based control of Fusarium graminearum infections through spraying of long dsRNAs involves a plant passage and is controlled by the fungal silencing machinery. PLoS Pathog. 2016;12(10):e1005901. 27737019 10.1371/journal.ppat.1005901 PMC5063301

[19] Timmons L, Fire A. Specific interference by ingested dsRNA. Nature. 1998;395(6705):854. 9804418 10.1038/27579

[20] Timmons L, Court DL, Fire A. Ingestion of bacterially expressed dsRNAs can produce specific and potent genetic interference in Caenorhabditis elegans. Gene. 2001;263(1–2):103–112. 11223248 10.1016/s0378-1119(00)00579-5

[21] Kamath RS. Genome-wide RNAi screening in Caenorhabditis elegans. Methods. 2003;30(4):313–321. 12828945 10.1016/s1046-2023(03)00050-1

[22] Zotti M, Dos Santos EA, Cagliari D, et al. RNA interference technology in crop protection against arthropod pests, pathogens and nematodes. Pest Manag Sci. 2018;74:1239–1250. 29194942 10.1002/ps.4813

[23] Whangbo JS, Hunter CP. Environmental RNA interference. Trends Genet. 2008;24:297–305. 18450316 10.1016/j.tig.2008.03.007

[24] Winston WM, Sutherlin M, Wright AJ, et al. Caenorhabditis elegans SID-2 is required for environmental RNA interference. Proc Nat Acad Sci. 2007;104(25):10565–10570. 17563372 10.1073/pnas.0611282104 PMC1965553

[25] McEwan DL, Weisman AS, Hunter CP. Uptake of extracellular double-stranded RNA by SID-2. Mol Cell. 2012;47(5):746–754. 22902558 10.1016/j.molcel.2012.07.014 PMC3488460

[26] Jose AM, Kim YA, Leal-Ekman S, et al. Conserved tyrosine kinase promotes the import of silencing RNA into Caenorhabditis elegans cells. Proc Nat Acad Sci. 2012;109(36):14520–145205. 10.1073/pnas.1201153109 PMC3437824 22912399

[27] Gao J, Zhao L, Luo Q, et al. An EHBP-1-SID-3-DYN-1 axis promotes membranous tubule fission during endocytic recycling. PLoS Genet. 2020;16(5):e1008763. 32384077 10.1371/journal.pgen.1008763 PMC7239482

[28] Imae R, Dejima K, Kage-Nakadai E, et al. Endomembrane-associated RSD-3 is important for RNAi induced by extracellular silencing RNA in both somatic and germ cells of Caenorhabditis elegans. Sci Rep. 2016;6(1):28198. 27306325 10.1038/srep28198 PMC4910058

[29] Winston WM, Molodowitch C, Hunter CP. Systemic RNAi in C. elegans requires the putative transmembrane protein SID-1. Science. 2002;295(5564):2456–2459. 11834782 10.1126/science.1068836

[30] Feinberg EH. Transport of dsRNA into cells by the transmembrane protein SID-1. Science. 2003;301(5639):1545–1547. 12970568 10.1126/science.1087117

[31] Shih JD, Fitzgerald MC, Sutherlin M, et al. 1 double-stranded RNA transporter is not selective for dsRNA length. RNA. 2009;15(3):384–390. 19155320 10.1261/rna.1286409 PMC2657005

[32] Shih JD, Hunter CP. SID-1 is a dsRNA-selective dsRNA-gated channel. RNA. 2011;17(6):1057–1065. 21474576 10.1261/rna.2596511 PMC3096038

[33] Jose AM, Garcia GA, Hunter CP. Two classes of silencing RNAs move between Caenorhabditis elegans tissues. Nat Struct Mol Biol. 2011;18(11):1184–1188. 21984186 10.1038/nsmb.2134 PMC3210371

[34] Tabara H, Grishok A, Mello CC. RNAi in C. elegans: soaking in the genome sequence. Science. 1998;282(5388):430–431. 9841401 10.1126/science.282.5388.430

[35] Hinas A, Wright AJ, Hunter CP. SID-5 is an endosome-associated protein required for efficient systemic RNAi in C. elegans. Curr Biol. 2012;22(20):1938–1943. 22981770 10.1016/j.cub.2012.08.020 PMC10518204

[36] Zhao Y, Holmgren BT, Hinas A. The conserved SNARE SEC-22 localizes to late endosomes and negatively regulates RNA interference in Caenorhabditis elegans. RNA. 2017;23(3):297–307. 27974622 10.1261/rna.058438.116 PMC5311485

[37] Palominos MF, Verdugo L, Gabaldon C, et al. Transgenerational diapause as an avoidance strategy against bacterial pathogens in caenorhabditis elegans. MBio. [Internet] 2017; 8(5). http://dx.doi.10.1128/mBio.01234-17 10.1128/mBio.01234-17 PMC5635688 29018118

[38] Franks DM, Izumikawa T, Kitagawa H, et al. C. elegans pharyngeal morphogenesis requires both de novo synthesis of pyrimidines and synthesis of heparan sulfate proteoglycans. Dev Biol. 2006;296:409–420. 16828468 10.1016/j.ydbio.2006.06.008

[39] Ho J, Tumkaya T, Aryal S, et al. Moving beyond P values: data analysis with estimation graphics. Nat Methods. 2019;16(7):565–566. 31217592 10.1038/s41592-019-0470-3

[40] Gardner MJ, Altman DG. Confidence intervals rather than P values: estimation rather than hypothesis testing. Br Med J. 1986;292(6522):746–750. 3082422 10.1136/bmj.292.6522.746 PMC1339793

[41] Zečić A, Dhondt I, Braeckman BP. The nutritional requirements of Caenorhabditis elegans. Genes Nutr. 2019;14:15. 31080524 10.1186/s12263-019-0637-7 PMC6501307

[42] Akay A, Jordan D, Navarro IC, et al. Identification of functional long non-coding RNAs in C. elegans. BMC Biol. 2019;17(1):14. 30777050 10.1186/s12915-019-0635-7 PMC6378714

[43] Boeck ME, Huynh C, Gevirtzman L, et al. The time-resolved transcriptome of C. elegans. Genome Res. 2016;26(10):1441–1450. 27531719 10.1101/gr.202663.115 PMC5052054

[44] Lands WE. Metabolism of glycerolipides; a comparison of lecithin and triglyceride synthesis. J Biol Chem. 1958;231:883–888. 13539023

[45] Lands WE. Metabolism of glycerolipids. 2. The enzymatic acylation of lysolecithin. J Biol Chem. 1960;235:2233–2237. 14413818

[46] Lands WE, Merkl I. Metabolism of glycerolipids. III. Reactivity of various acyl esters of coenzyme A with alpha’-acylglycerophosphorylcholine, and positional specificities in lecithin synthesis. J Biol Chem. 1963;238:898–904. 13928489

[47] Merkl I, Lands WE. Metabolism of glycerolipids. IV. Synthesis of phosphatidylethanolamine. J Biol Chem. 1963;238:905–906. 13935003

[48] Goetzl EJ, An S. Diversity of cellular receptors and functions for the lysophospholipid growth factors lysophosphatidic acid and sphingosine 1-phosphate. Faseb J. 1998;12(15):1589–1598. 9837849

[49] Kume N, Gimbrone MA Jr. Lysophosphatidylcholine transcriptionally induces growth factor gene expression in cultured human endothelial cells. J Clin Invest. 1994;93:907–911. 7509351 10.1172/JCI117047 PMC293967

[50] Gao AW, Chatzispyrou IA, Kamble R, et al. A sensitive mass spectrometry platform identifies metabolic changes of life history traits in C. elegans. Sci Rep. 2017;7(1):2408. 28546536 10.1038/s41598-017-02539-w PMC5445081

[51] Appleford PJ, Griffiths M, Yao SYM, et al. Functional redundancy of two nucleoside transporters of the ENT family (CeENT1, CeENT2) required for development of Caenorhabditis elegans. Mol Membr Biol. 2004;21(4):247–259. 15371014 10.1080/09687680410001712550

[52] Xiao G, Wang J, Tangen T, et al. A novel proton-dependent nucleoside transporter, CeCNT3, from Caenorhabditis elegans. Mol Pharmacol. 2001;59(2):339–348. 11160871 10.1124/mol.59.2.339

[53] Mörck C, Pilon M. C. elegans feeding defective mutants have shorter body lengths and increased autophagy. BMC Dev Biol. 2006;6(1):39. 16884547 10.1186/1471-213X-6-39 PMC1559592

[54] Shtonda BB, Avery L. Dietary choice behavior in Caenorhabditis elegans. J Exp Biol. 2006;209:89–102. 16354781 10.1242/jeb.01955 PMC1352325

[55] Uppaluri S, Brangwynne CP. A size threshold governs Caenorhabditis elegans developmental progression. Proc Biol Sci. 2015;282:20151283. 26290076 10.1098/rspb.2015.1283 PMC4632629

[56] Macedo F, Martins GL, Luévano-Martínez LA, et al. Lipase-like 5 enzyme controls mitochondrial activity in response to starvation in Caenorhabditis elegans. Biochim Biophys Acta Mol Cell Biol Lipids. 2020;1865(2):158539. 31676440 10.1016/j.bbalip.2019.158539

[57] Szewczyk NJ, Kozak E, Conley CA. Chemically defined medium and Caenorhabditis elegans. BMC Biotechnol. 2003;3(1):19. 14580264 10.1186/1472-6750-3-19 PMC270041

[58] MacNeil LT, Watson E, Arda HE, et al. Diet-induced developmental acceleration independent of TOR and insulin in C. elegans. Cell. 2013;153:240–252. 23540701 10.1016/j.cell.2013.02.049 PMC3821073

[59] Byerly L, Cassada RC, Russell RL. The life cycle of the nematode Caenorhabditis elegans. I. Wild-type growth and reproduction. Dev Biol. 1976;51(1):23–33. 988845 10.1016/0012-1606(76)90119-6

[60] Woodruff GC, Willis JH, Phillips PC. Dramatic evolution of body length due to postembryonic changes in cell size in a newly discovered close relative of Caenorhabditis elegans. Evol Lett. 2018;2:427–441. 30283693 10.1002/evl3.67 PMC6121821

[61] Brenner S. The genetics of Caenorhabditis elegans. Genetics. 1974;77:71–94. 4366476 10.1093/genetics/77.1.71 PMC1213120

[62] Flemming AJ, Shen ZZ, Cunha A, et al. Somatic polyploidization and cellular proliferation drive body size evolution in nematodes. Proc Natl Acad Sci U S A. 2000;97(10):5285–5290. 10805788 10.1073/pnas.97.10.5285 PMC25820

[63] Johnstone IL. Cuticle collagen genes. Expression in Caenorhabditis elegans. Trends Genet. 2000;16(1):21–27. 10637627 10.1016/s0168-9525(99)01857-0

[64] Hibshman JD, Hung A, Ryan Baugh L. Maternal diet and insulin-like signaling control intergenerational plasticity of progeny size and starvation resistance. PLoS Genet. 2016;12(10):e1006396. 27783623 10.1371/journal.pgen.1006396 PMC5081166

[65] So S, Miyahara K, Ohshima Y. Control of body size in C. elegans dependent on food and insulin/IGF-1 signal: body size control in C. elegans. Genes Cells. 2011;16:639–651. 21501345 10.1111/j.1365-2443.2011.01514.x

[66] McCulloch D. Body size, insulin/IGF signaling and aging in the nematode Caenorhabditis elegans. Exp Gerontol. 2003;38(1–2):129–136. 12543270 10.1016/s0531-5565(02)00147-x

[67] Nuez I, Félix M-A. Evolution of susceptibility to ingested double-stranded RNAs in Caenorhabditis nematodes. PLoS One. 2012;7(1):e29811. 22253787 10.1371/journal.pone.0029811 PMC3256175

[68] Khajuria C, Ivashuta S, Wiggins E, et al. Development and characterization of the first dsRNA-resistant insect population from western corn rootworm, Diabrotica virgifera virgifera LeConte. PLoS One. 2018;13(5):e0197059. 29758046 10.1371/journal.pone.0197059 PMC5951553

[69] Liu H, Wang X, Wang H-D, et al. Escherichia coli noncoding RNAs can affect gene expression and physiology of Caenorhabditis elegans. Nat Commun. 2012;3(1):1073. 23011127 10.1038/ncomms2071 PMC3658002

[70] Akay A, Sarkies P, Miska EA. E. coli OxyS non-coding RNA does not trigger RNAi in C. elegans. Sci Rep. 2015;5(1):9597. 25873159 10.1038/srep09597 PMC4397834

[71] Samuel BS, Rowedder H, Braendle C, et al. Caenorhabditis elegans responses to bacteria from its natural habitats. Proc Natl Acad Sci U S A. 2016;113(27):E3941–9. 27317746 10.1073/pnas.1607183113 PMC4941482

[72] Félix M-A, Braendle C. The natural history of Caenorhabditis elegans. Curr Biol. 2010;20(22):R965–9. 21093785 10.1016/j.cub.2010.09.050

[73] Frézal L, Félix M-A. C. elegans outside the Petri dish. Elife. [[Internet] 2015; 4. Available from]: https://www.ncbi.nlm.nih.gov/pubmed/25822066 10.7554/eLife.05849 PMC4373675 25822066

[74] Melo JA, Ruvkun G. Inactivation of conserved C. elegans genes engages pathogen- and xenobiotic-associated defenses. Cell. 2012;149(2):452–466. 22500807 10.1016/j.cell.2012.02.050 PMC3613046

[75] Kaletsky R, Moore RS, Vrla GD, et al. C. elegans “reads” bacterial non-coding RNAs to learn pathogenic avoidance [Internet]. bioRxiv. 2020. cited 2020 310. 2020.01.26.920322. https://www.biorxiv.org/content/10.1101/2020.01.26.920322v1 10.1038/s41586-020-2699-5 PMC8547118 32908307

[76] Paix A, Folkmann A, Seydoux G. Precision genome editing using CRISPR-Cas9 and linear repair templates in C. elegans. Methods. 2017;121:86–93. 28392263 10.1016/j.ymeth.2017.03.023 PMC6788293

[77] Dobin A, Davis CA, Schlesinger F, et al. STAR: ultrafast universal RNA-seq aligner. Bioinformatics. 2013;29:15–21. 23104886 10.1093/bioinformatics/bts635 PMC3530905

[78] Liao Y, Smyth GK, Shi W. featureCounts: an efficient general purpose program for assigning sequence reads to genomic features. Bioinformatics. 2013;30(7):923–930. 24227677 10.1093/bioinformatics/btt656

[79] Anders S, Huber W. Differential expression analysis for sequence count data. Genome Biol. 2010;11(10):R106. 20979621 10.1186/gb-2010-11-10-r106 PMC3218662

[80] Benjamini Y, Hochberg Y. Controlling the false discovery rate: A practical and powerful approach to multiple controlling the false discovery rate. Source JR Stat Soc Ser BJR Stat Soc Ser BMethodological). JR Stat Soc B. 1995;57:289–300.

[81] Angeles-Albores D, Lee RYN, Chan J, et al. Two new functions in the wormbase enrichment suite, microPublication Biol, 2018 10.17912/W25Q2N PMC7255849 32550381

[82] Virtue S, Petkevicius K, Moreno-Navarrete JM, et al. Peroxisome proliferator-activated receptor γ2 controls the rate of adipose tissue lipid storage and determines metabolic flexibility. Cell Rep. 2018;24(8):2005–12.e7. 30134163 10.1016/j.celrep.2018.07.063 PMC6113930

