# Supplementary material for: SID-2 negatively regulates development likely independent of nutritional dsRNA uptake
Source: RNA Biol. 2020 Oct 12;18(6):888–99. doi: 10.1080/15476286.2020.1827619 (PMC8081039; doi:10.1080/15476286.2020.1827619)

A

embryo transcripts

Term	Observed	Enrichment Fold Change	Q value
larval physiology variant	8	6.6	0.00093
dauer constitutive	3	17	0.0033
dauer metabolism variant	5	5.9	0.017
fat content increased	3	8.6	0.025
male nervous system morphology variant	2	14	0.025
Variant	25	1.9	0.025

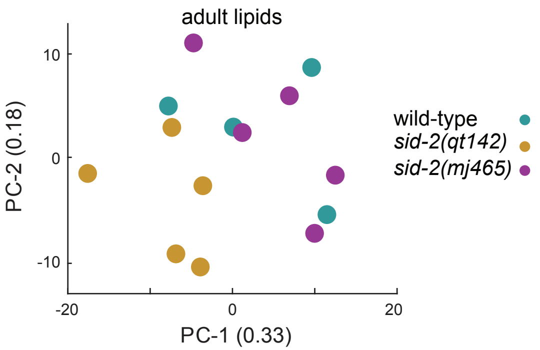

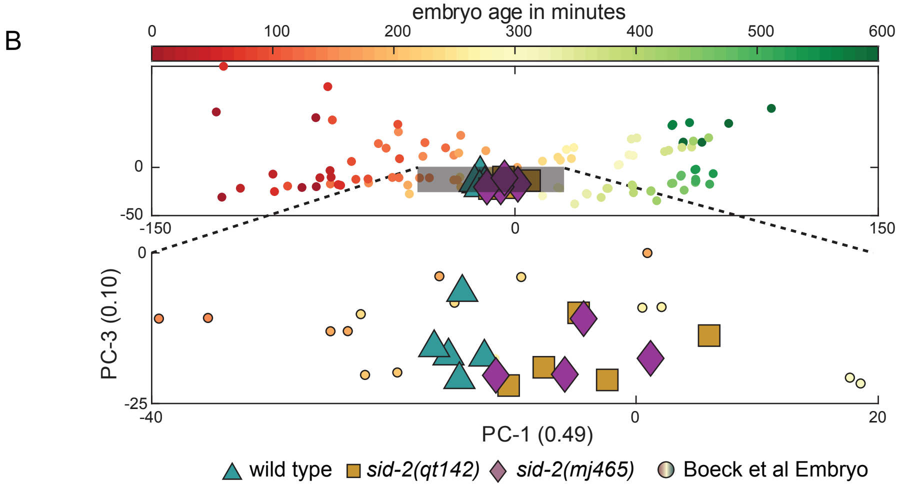

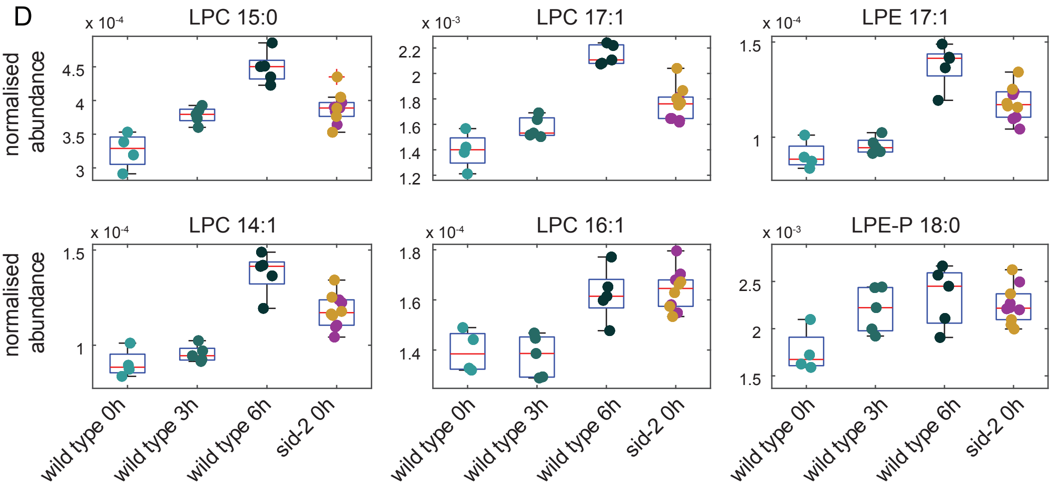

Supplement: Supplemental Material [file KRNB_A_1827619_SM9129.zip › Supplementary information/FigureS3.pdf]
