# Supplementary figures and images for: SID-2 negatively regulates development likely independent of nutritional dsRNA uptake
Source: RNA Biol. 2020 Oct 12;18(6):888–99. doi: 10.1080/15476286.2020.1827619 (PMC8081039; doi:10.1080/15476286.2020.1827619)

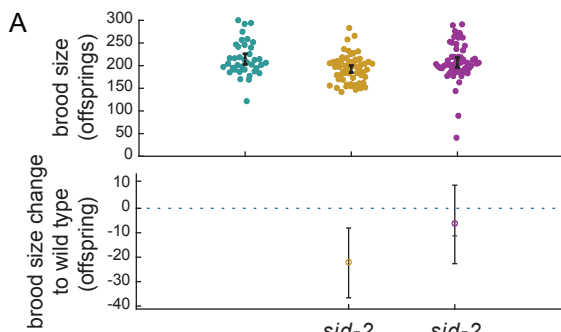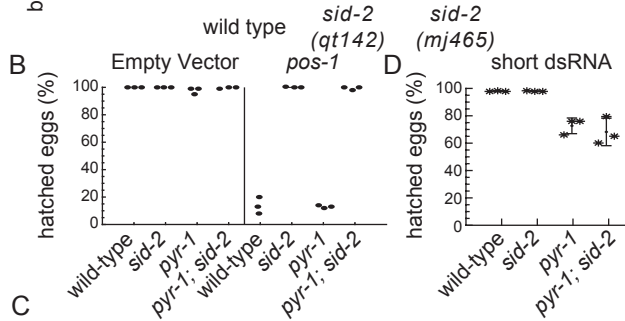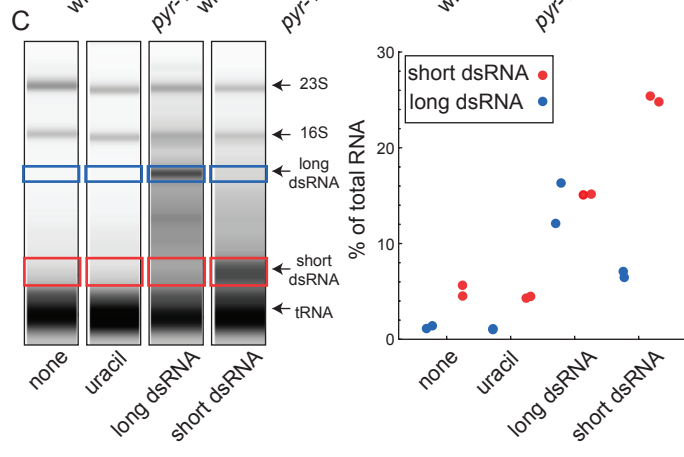

Supplement: Supplemental Material [file KRNB_A_1827619_SM9129.zip › Supplementary information/FigureS1.pdf]

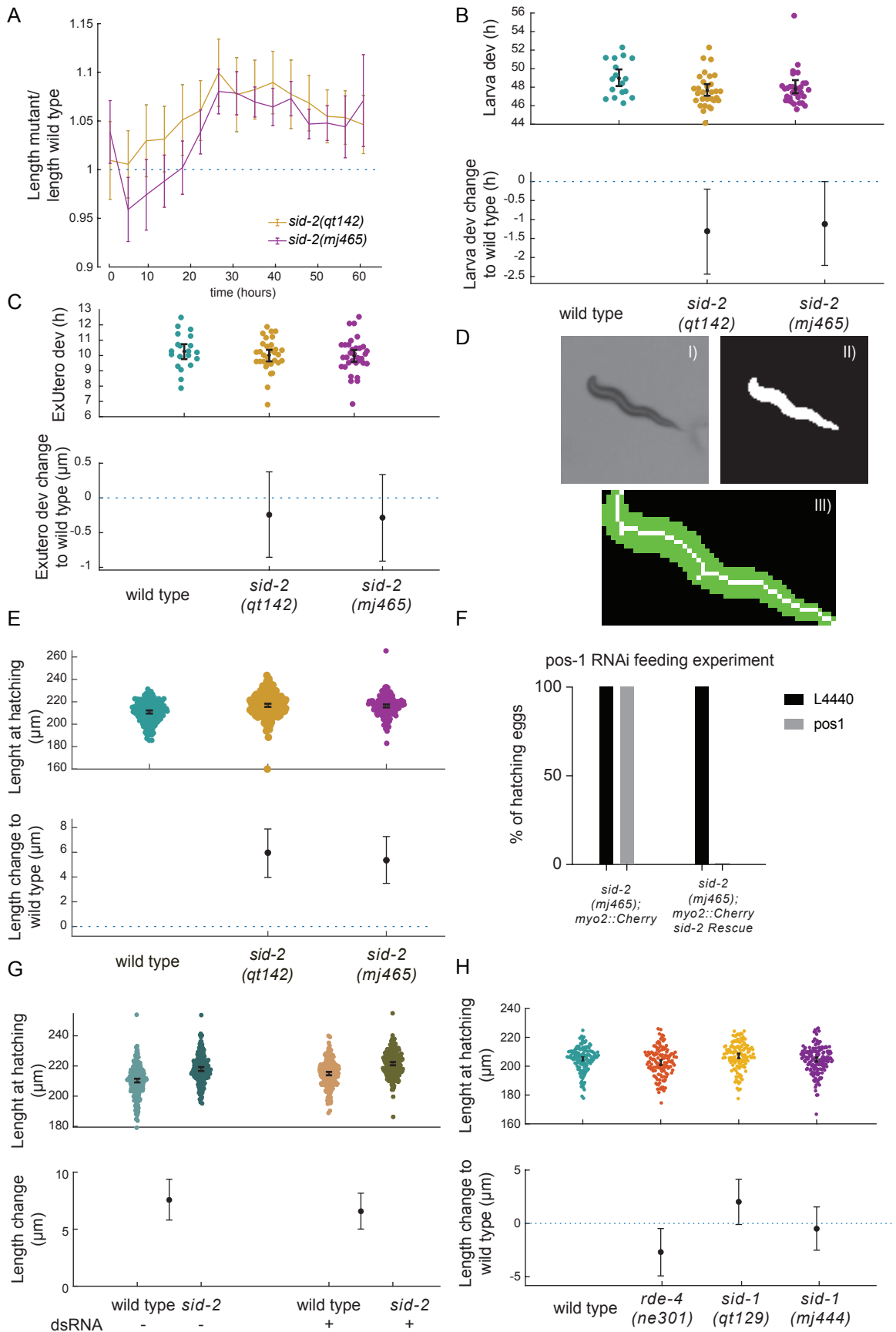

Supplement: Supplemental Material [file KRNB_A_1827619_SM9129.zip › Supplementary information/FigureS2.pdf]
